# Fellow-Workers and Their Doings

**Published:** 1914-04-18

**Authors:** 


### ROUND THE WORLD.

### Fellow-Workers and their Doings.

[The Editor Invites Early Information and Contributions Marked "Fellow-Workers."]

Mr. Percy Dunn, F.R.C.S., senior ophthalmic surgeon at the West London Hospital, in a recent communication, has boldly stated what many feel—namely, that in the modern experimental method of cancer research upon mice, investigators are leaving the high-road for a by-path. He is convinced that not only cancer but many other diseases are due to a perverted metabolism. He himself finds thyroid extract to have a prompt effect in keratitis punctata. Mr. Dunn is strongly in favour of the establishment of a bio-clinical laboratory in every large hospital for the investigation of the endogenous origins of disease.

Mr. Gwynne E. O. Williams, M.D., F.R.C.S., has been appointed assistant surgeon on the staff of University College Hospital to fill the vacancy occasioned by the resignation of Mr. Bilton Pollard and subsequent promotions. Mr. Williams has lately been performing the duties of surgical registrar at the hospital (previous to which he held various resident appointments there) and assistant medical officer to the Lewisham Infirmary.

Dr. H. Ridley Prentice, M.B., M.R.C.P., one of the new physicians at the *Dreadnought* Hospital at Greenwich, is a "Bart.'s" man, and has been giving particular attention to nervous diseases, having served as resident medical officer to the National Hospital for Paralysis and Epilepsy, and has also translated Professor Romer's book on "Epidemic Infantile Paralysis."

Dr. Ernest Ward, M.D. Cantab., F.R.C.S., is a practitioner at Stockton-on-Tees whose keenness of observation and scientific habits have not become blunted by general work. Since qualification he has contributed frequent papers on his own cases to the medical press. A method of conserving injured fingers, termed "sponge education," was widely read in the *Medical Review* of 1911. His latest communication on multiple warts in pregnancy (*British Journal of Dermatology*) will be of interest to most men in general practice. Dr. Ward is a prime mover in the recently organised scheme of co-ordinated research by general practitioners.

The activities of a busy "G.P." are illustrated by Dr. Guy Wyon, B.Sc. Lond., M.B. Edin., who, besides running a busy panel practice in Bow, is an enthusiastic politician in the cause of a State medical service. Moreover, much of his spare time is given up to pathological research, Dr. Wyon's present interest in this field being the perfection of a technique for Abderhalden's serum test of pregnancy. He is at present getting consistent results with this test, and believes it has a field of useful application in medical work.

An extension of his leave of absence has lately been granted to Mr. Francis Jaffrey, surgeon to St. George's Hospital. It is understood that his health is not yet as good as his many friends could wish it. Mr. Jaffrey is second in seniority upon the surgical staff at St. George's to Mr. G. R. Turner, and is also surgeon to the Belgrave Hospital for Children. His burly figure and genial ways will be missed at Hyde Park Corner, and we trust that the severance is only temporary.

Dr. Hinds Howell, M.D., F.R.C.P., who has lately devoted much time to the study of nervous diseases, has endeavoured to assist practitioners in gaining a better understanding of the painful conditions to which the diagnosis of "neuritis" is commonly attached. As assistant to the National Hospital for Paralysis and Epilepsy, as well as in the office of registrar at "Bart.'s," Dr. Hinds Howell has had plenty of opportunities of doing advanced work in neurology. A suggestion which he has made recently is to the effect that those trophic changes frequently associated with chronic nervous disorders, and by many supposed to be due to interference with special untrophic nerves, are probably only the consequences of an altered blood supply due to a disturbance of the sympathetic system.

Mr. Charles P. Childe, M.R.C.P. Edin., F.R.C.S. Eng., deserves credit for his efforts to decrease the mortality of cancer in the British Isles Realising that in our present want of knowledge as to the etiology of malignant tumours and inability to deal with them effectually other than by an operation in the early stages, Mr. Childe set out to educate both the rank and file of the profession and the intelligent public as to the urgency of having every new growth, however small, submitted to expert examination as soon as possible. His interesting book, entitled "The Control of a Scourge," published some years ago, clearly explains the points at issue. Lately, as Vice-Chairman on the Health Committee at Portsmouth, Mr. Childe has been instrumental in carrying out several important measures having in view the prevention of much suffering from cancer. One of these is a movement to publish regularly information that will lead the general public to understand that where cancer is taken in the early stages there is a reasonable possibility of its being thoroughly removed, and, moreover, giving various hints as to likely signs of cancer in its beginnings, urging upon all who know such signs to seek medical advice at once. Mr. Childe is an old student of the Cape of Good Hope University, and also of King's College, London; for some years he has been an active member of the staff of the Royal Portsmouth Hospital.

Dr. F. Vincent Denne, who has been appointed dental surgeon to the West End Hospital for Nervous Diseases in Welbeck Street, is dental surgeon to the London County Council School Clinic at the St. Marylebone General Dispensary, which is practically next door to the institution where he has taken up his new duties. Besides holding the dental diplomas of the Royal Colleges, Dr. Denne holds also their conjoint qualification, and is an M.D. of Brussels University.

Dr. Charles Edward Kynaston Herapath, the new medical registrar to the Bristol Royal Infirmary, is a student of the local university, and subsequently to his qualification in 1907 held several resident appointments at the institution at which he has now once more been given an important office. Dr. Kynaston Herapath is surgeon to the Bristol Dispensary, and became an M.D. of London University four years ago.

